# Editorial Announcements

**Published:** 1912-02

**Authors:** 


### BUFFALO MEDICAL JOURNAL

**A Monthly Review of Medicine and Surgery**

*EDITOR*

A. L. BENEDICT.

All communications, whether of a literary or business nature, books for review and exchanges, should be addressed to the editor, 354 Franklin Street, Buffalo, N. Y Please make personal or telephonic calls before 1 p. m

The Buffalo Medical Journal will publish regularly, full transactions of any professional organization in western New York at cost. Personal notices, brief reports of society proceedings, and the like, are always welcome and will be published without charge.

Subscriptions may commence at any time. $2.00 per year in advance,

Vol. LXVII. FEBRUARY, 1912 No. 7

*Which part of a medical meeting do you enjoy most, learning a new test, treatment or scientific fact; fighting over an amendment; or the collation?*

### Editorial Announcements.

The interesting and ideally brief article "Penetrating Wound of the Orbit with Dislodgement of the Vomer," in the January issue, was by Dr. H. W. Cowper. The omission was supplied on both sets of proof but, somehow persisted.

Physicians have, from time to time, received announcements of ordinances and laws regarding the reporting of disease, filing of certificates, etc. Many other legal requirements exist of which the younger physicians have never been informed and which the older ones may have forgotten. Dr. Francis E. Fronczak is preparing a careful tabulation of national, state and local requirements of this general nature, for the guidance of physicians. We expect to publish the first installment in the March issue.

